# Long-Term Effects of Neurofeedback and Hyperbaric Oxygen Therapy on Traumatic Brain Injury: A Principal Component Analysis (PCA)-Based Secondary Analysis

**DOI:** 10.7759/cureus.74305

**Published:** 2024-11-23

**Authors:** Tami Peterson, JeAnnah AbouAssaly, Sheila Burgin, Robert Sherwin, Frederick Strale

**Affiliations:** 1 Hyperbaric Oxygen Therapy, The Oxford Center, Brighton, USA; 2 Neurofeedback, The Oxford Center, Brighton, USA; 3 Hyperbaric Oxygen Therapy, Wayne State University School of Medicine, Detroit, USA; 4 Biostatistics, The Oxford Center, Brighton, USA

**Keywords:** hyperbaric oxygen therapy (hbot), neurofeedback therapy, nfb, pca, pre-post treatment, principal components analysis, qeeg, quantitative electroencephalogram, severe traumatic brain injury, traumatic brain injury(tbi)

## Abstract

Severe traumatic brain injury (TBI) poses significant public health challenges, but treatments like neurofeedback and hyperbaric oxygen therapy (HBOT) show promise in aiding recovery. Neurofeedback enhances brain healing through operant conditioning, while HBOT increases cerebral oxygenation, supporting cognitive recovery. A 33-year-old woman, after suffering a severe TBI in 2018 and a long rehabilitation, began HBOT and neurofeedback in late 2021. By early 2022, she demonstrated significant cognitive, emotional, and social improvements. After numerous sessions, a June 2024 quantitative electroencephalogram (qEEG) revealed substantial brain recovery, with marked gains (Peterson et al.’s initial study) in daily functioning and specific tasks. This secondary analysis conducted in November 2024 used principal component analysis (PCA) on the initial pretest, posttest, and difference score data from the treatment period to explore the neurophysiological effects of the combined therapies. The results showed notable factor structure differences in brainwave patterns and electrode activity from the pretest to the posttest. The simpler structure observed in pretests evolved into a more complex factor structure with posttest and difference scores, indicating neurophysiological adaptations due to the interventions. This study’s PCA findings align with the post-treatment qEEG statistical results conducted in June 2024 (Peterson et al.’s initial study), which identified moderate to large improvement effect sizes in the patient’s brain’s average frequency band parameters (g = 0.612) and small to moderate effect sizes on 19 electrode placement outcomes (uV² g = 0.339 and Hz g = 0.333). The June 2024 results indicated significant progress over a 31-month treatment period. In June 2024, the Disability Rating Scale (DRS) and the Glasgow Outcome Scale Extended (GOSE) showed substantial improvements in cognitive abilities such as feeding, toileting, grooming, and communication skills. According to the qEEG effect sizes, as well as DRS and GOSE scores from the pretest (2021) and posttest (2024), the patient demonstrated meaningful gains in brain recovery and overall quality of life. The cognitive improvements identified in the June 2024 Wilcoxon test were further corroborated by the factor structure analysis conducted in the November 2024 PCA. This alignment between the Wilcoxon test results and the PCA findings underscores the robustness of the observed cognitive gains, providing a comprehensive validation of the patient’s progress. The consistency across these distinct analytical methods highlights the significant strides made in cognitive function, reinforcing the efficacy of the treatment regimen over the observed period.

## Introduction

Traumatic brain injury (TBI) is the most common neurological disorder and poses a significant public health challenge. Increasingly, TBI is recognized not only as an acute condition but also as a chronic disease with long-term effects, including an elevated risk of neurodegeneration later in life [1]. It remains a leading cause of global mortality and disability [2]. TBI symptoms vary depending on the severity and location of the injury and can include cognitive decline, physical impairments, mood and emotional disturbances, and sleep problems.

In 2020, there were approximately 214,110 TBI-related hospitalizations, and in 2021, there were 69,473 TBI-related deaths, averaging over 586 hospitalizations and 190 deaths per day. These numbers do not include TBIs treated in emergency rooms, primary or urgent care, or untreated cases. Despite significant research on TBIs, further studies are needed to develop more effective clinical treatments for neurological conditions [3,4].

TBI neurofeedback therapy

Using operant conditioning, neurofeedback enables patients to gain real-time control over their physiological responses, enhancing well-being and alleviating symptoms. Typically incorporating quantitative electroencephalogram (qEEG) data, neurofeedback sessions provide feedback through visual and auditory cues under professional supervision. Patients learn to regulate their responses to cognitive, emotional, or physical stimuli, improving their awareness and control over these reactions in daily life [5-7].

Research has shown the benefits of neurofeedback for TBI recovery. For example, Chen et al. found that neurofeedback improved memory [8,9], attention [10], and motor skills integration [11] in TBI patients using qEEG-based positive feedback protocols [12-14]. Gupta et al.’s study with TBI patients demonstrated significant reductions in post-concussion symptoms and normalized qEEG readings, suggesting neurofeedback as a potential treatment for TBI [15]. Munivenkatappa et al.’s protocol enhanced mental speed, memory, and recall in young patients with moderate TBI [16,17]. However, Rostami et al.’s research showed mixed results, with no significant improvements in memory or concentration [18].

In 2021, Arroyo-Ferrer et al. compared neurofeedback to traditional cognitive rehabilitation in a 20-year-old TBI patient, finding that neurofeedback improved delayed memory while conventional methods benefited short-term memory. These studies collectively highlight neurofeedback’s potential as an alternative or complementary therapy for cognitive rehabilitation in TBI patients [19].

Hyperbaric oxygen therapy (HBOT) for TBI

HBOT is being investigated as a treatment for TBI, with the potential to enhance cognitive function and the body’s healing process by increasing blood oxygen levels. Research indicates that HBOT can alleviate cognitive symptoms in TBI patients by delivering oxygen more deeply into damaged brain tissues, promoting recovery [20,21].

A Cochrane Review found that HBOT can reduce mortality risk and improve coma outcomes in TBI patients, although it does not significantly affect long-term quality of life. Other studies have shown that 40 HBOT sessions at 1.5 atmospheres can significantly improve cognitive and symptomatic outcomes in patients with mild TBI and persistent post-concussion syndrome (PPCS). Additionally, repeated HBOT sessions may promote neuroplasticity, improve brain perfusion, and enhance cognitive functions such as memory, processing speed, and motor skills, even years after the injury [22].

Clinical trials with moderate TBI patients experiencing prolonged PPCS have shown that HBOT can improve quality of life, cognitive function, and brain activity [23,24]. Retrospective studies of chronic TBI cases have also reported significant cognitive improvements, particularly in memory and attention. Overall, HBOT shows promise in supporting neuroplasticity and enhancing the quality of life for TBI patients at various stages of recovery [25-27].

Integrating neurofeedback and HBOT for treating TBI

White et al. [4] reported on a case involving a 26-year-old man who suffered a severe TBI from a car accident. This injury resulted in closed head trauma to the left temporal region, with a coup-contrecoup effect affecting the frontal area. After a craniotomy and a 26-day coma, he regained consciousness and was moved to a specialized brain injury rehabilitation center, where he received physical, speech, and occupational therapy. Eight months later, he was discharged but continued to experience challenges with speech, mobility, spasticity, cognition, and posttraumatic epilepsy.

His family sought HBOT from a physician in Louisiana. After completing 165 sessions, they also incorporated neurofeedback therapy in March 2019. This combined approach of HBOT and neurofeedback showed improvements in brain plasticity and functionality in affected areas and alleviated symptoms such as short-term memory issues, personality changes, language difficulties, executive function impairments, and seizure frequency [4].

This case underscores the potential effectiveness of combining HBOT and neurofeedback in treating severe TBI, particularly given the persistent deficits often associated with such injuries. White et al.’s study is one of the few to investigate this combined method, highlighting the necessity for further research into long-term neurological treatment strategies for severe TBI [4].

Peterson et al. documented the case of a 33-year-old female runner who was struck by a car traveling at 40 mph and thrown 30 feet, resulting in a severe TBI and a seven-week coma. Following seven months of intensive rehabilitation, she began HBOT and neurofeedback treatments in November 2021, as recommended by her neuropsychiatrist. By January 2022, these therapies led to improvements in her cognitive function, sleep quality, conversation skills, emotional regulation, and relationships [28].

As of December 2023, after completing 195 neurofeedback sessions and over 300 HBOT sessions, she reported further enhancements in various cognitive and emotional areas, as well as in daily activities such as feeding, toileting, grooming, and communication. A post-treatment qEEG conducted in June 2024 indicated moderate to large improvements in her brain’s average frequency band parameters (g = 0.612) and small to moderate effects on 19 electrode placement outcomes (uV² g = 0.339 and Hz g = 0.333) [28].

These results reflect significant progress over a 31-month treatment period. Objective measures, including the Disability Rating Scale (DRS) and the Glasgow Outcome Scale Extended (GOSE), showed substantial improvements in cognitive abilities such as feeding (p = 0.046), toileting (p = 0.046), grooming (p = 0.046), and communication skills (p = 0.046). According to the qEEG effect sizes, as well as DRS and GOSE scores from the pretest (2021) and posttest (2024), the patient demonstrated meaningful gains in brain recovery and overall quality of life [28].

This secondary analysis aims to utilize the existing dataset from Peterson et al.’s original case study, published via The Oxford Center (TOC) enhancing the initial findings through principal component analysis (PCA) [28]. This advanced statistical method will be applied to explore the brainwave-specific factor structure, including delta, theta, alpha, beta1, beta2, and gamma frequencies (Hz) and power measurements (uV²) from the 19 cortical scalp sites. The goal is to identify distinct brainwave patterns by analyzing pretest, posttest, and difference scores, thus providing deeper insights into the neurophysiological and statistical aspects of the treatment effects observed with neurofeedback and HBOT. To date, no studies have successfully explored this approach.

## Case presentation

Methods

Participant and Procedure

The patient agreed to participate in this study, and her caregivers provided informed consent for both the neurofeedback and HBOT treatments, as well as for the research use of the generated qEEG data. She received neurofeedback and HBOT treatments at TOC located in Brighton and Troy, Michigan, USA. These outpatient facilities offer a variety of services for different biopsychosocial conditions [28]. A baseline (pretest) qEEG was conducted on November 10, 2021, prior to the commencement of neurofeedback treatments. During the qEEG data collection, the patient wore a Channel EEG Cap equipped with 19 scalp electrodes arranged according to the International 10/20 System (Fp1, Fp2, F7, F3, Fz, F4, F8, T7, C3, Cz, C4, T8, P7, P3, Pz, P4, P8, O1, and O2), which included ear connectors. The qEEG action potentials from the 19 channels were recorded for 20 to 30 minutes using the Discovery 24-channel qEEG amplifier, ensuring impedances remained below 10 KOhms. The qEEG data was then uploaded into WinEEG for analysis, where artifacts were identified and removed. The results included power measurements in microvolts squared (uV²) and hertz (Hz) for Delta, Theta, Alpha, Beta1, Beta2, and Gamma frequencies [28].

Inclusion and Exclusion Criteria

This research utilized a single research subject, a 33-year-old female who had sustained a severe TBI, to investigate the effects of combined HBOT and neurofeedback therapy. The scientific rationale for selecting this subject was based on her specific medical history and the unique challenges posed by her condition. Inclusion criteria for this study included a diagnosis of severe TBI, age within the range of 18 to 45 years, and a willingness to participate in both HBOT and neurofeedback sessions. The subject’s lack of prior significant neurological disorders, stable medical condition, and absence of contraindications for HBOT were also crucial factors for inclusion.

Exclusion criteria were established to ensure the safety and validity of the research findings. These criteria included any history of psychiatric disorders that could influence cognitive assessments, pregnancy, or any other medical conditions that might contraindicate HBOT, such as certain lung diseases. Additionally, individuals currently undergoing other neurorehabilitative therapies or medications that affect neurological function were excluded to maintain the integrity of the therapeutic interventions being tested.

Devices for Neurofeedback Treatments

The Discovery 24-channel qEEG amplifier is a physiological monitoring system designed to measure qEEG, direct current, and slow cortical potentials. It features 24 channels for qEEG biofeedback, with 22 channels connected through a standard electrode cap and two differential input channels. The device captures 1,024 samples per second at a 24-bit resolution, with an amplifier bandwidth ranging from DC (0.000 Hz) to 80 Hz. Its lightweight and portable design makes it suitable for use with laptops in both remote training and clinical environments, and it is powered entirely via USB, ensuring client safety without the need for batteries [29].

The WaveGuard Connect qEEG cap is equipped with soft silicone electrode cups that have concealed wiring and high-density connectors, ensuring safe and efficient use. It is particularly suited for routine diagnostics, providing high signal quality through reliable and long-lasting tin electrode sensors. The electrodes are pre-arranged according to the international 10/20 system, allowing for quick setup - typically completed within 10 minutes. The cap is compatible with major qEEG amplifiers and includes a default D-SUB 25 connector for straightforward connection, with an optional adapter available for third-party devices [30].

WinEEG is an advanced software solution for processing and analyzing qEEG and event-related potentials (ERPs) data. It supports the import of various file formats and can handle up to 256 recording channels, including event markers for ERP studies. The software offers quick access to recordings, artifact correction, and spectral analysis. It features capabilities for multichannel spectral analysis, brain mapping, coherence analysis, and wavelet analysis of ERPs. Additionally, WinEEG can integrate with Low-Resolution Electromagnetic Tomography (LORETA) software for 3D mapping and source localization, allowing users to export data in multiple formats for further statistical analysis [31].

Together, these qEEG technologies significantly enhance the monitoring, feedback, and analysis of brain activity, supporting clinical assessments and research in neurological disorders [29-31].

Neurofeedback Procedures

The neurofeedback training protocol primarily focused on the brain’s left hemisphere, the area affected by the injury. However, it also took into account the potential impact on interconnected brain regions, recognizing the complexity of brain networks. The protocol was tailored based on the patient’s qEEG readings. The main objective was to increase the power of alpha and beta brainwaves while reducing the amplitude of theta and delta waves. This approach was based on the belief that such changes in brainwave activity could support cognitive recovery [28].

The patient participated in neurofeedback training two to three times a week, with each session lasting 30 to 60 minutes. The schedule was flexible, allowing for breaks during holidays and personal commitments. Each session included computer games, animations, and sounds that responded to the patient’s brainwave activity. This interactive setup provided real-time feedback, encouraging the patient to consciously adjust their brainwave patterns through visual or auditory cues. The training involved cognitive tasks that required attention, memory, and executive function, reinforcing desired brainwave patterns to promote the restoration of healthy cognitive processes [28].

qEEG Posttest Assessments

On June 6, 2024, a posttest qEEG was conducted to ensure consistency and allow for comparison with earlier measurements. The data collection involved fitting the patient with a Channel qEEG Cap featuring 19 channels arranged according to the International 10/20 System. The electrode placements included Fp1, Fp2, F7, F3, Fz, F4, F8, T7, C3, Cz, C4, T8, P7, P3, Pz, P4, P8, O1, and O2, along with ear connectors to enhance signal quality. qEEG action potentials were continuously recorded using the Discovery 24-channel qEEG amplifier [28].

During the recording session, electrode impedances were carefully maintained below 10 KOhms to ensure high-quality data acquisition. The collected qEEG data were then uploaded into WinEEG software for comprehensive analysis, which included identifying and eliminating artifacts to ensure data accuracy. This analysis provided detailed insights into various brainwave activities, such as Delta, Theta, Alpha, Beta-1, Beta-2, and Gamma frequencies. These metrics offered valuable information about the patient’s neural function and enabled a thorough evaluation of changes following the neurofeedback and HBOT treatments that began in 2021 [28].

qEEG Codes for Power (uV²) and Frequency (Hz)

In qEEG, various abbreviations denote parameters related to brainwave activity, providing valuable insights into cognitive functions and potential abnormalities. DuV² (Delta microvolts squared) measures the power of delta waves (0.5-4 Hz), associated with deep sleep and restorative processes [28], while Dhz (Delta Hertz) represents their frequency [28]. TuV² (Theta microvolts squared) evaluates the power of theta waves (4-8 Hz), linked to meditation, early sleep stages, learning, and memory [28], with Thz (Theta Hertz) indicating their frequency [28]. AuV² (Alpha microvolts squared) measures alpha wave power (8-13 Hz), associated with relaxation, calmness, and attention [28], and Ahz (Alpha Hertz) denotes their frequency [28]. BuV² (Beta microvolts squared) captures the power of beta waves (13-30 Hz), associated with active thinking and focus [28], while Bhz (Beta Hertz) represents their frequency [28]. For higher-frequency beta waves (20-30 Hz), B2uV² (Beta2 microvolts squared) and B2hz (Beta2 Hertz) measure power and frequency, respectively, reflecting heightened alertness and focus [28]. Lastly, GuV² (Gamma microvolts squared) quantifies the power of gamma waves (30-100 Hz), associated with advanced cognitive functions such as learning and memory [28], while Ghz (Gamma Hertz) represents their frequency [28]. These metrics facilitate the assessment of brain activity and help identify underlying neurological patterns.

HBOT Procedures

From November 2021 to June 2024, a Class B monoplace hyperbaric chamber (Sechrist 3300H, Sechrist Industries, Inc., Anaheim, California, USA) was used at TOC in Brighton, MI, USA to provide HBOT to a patient diagnosed with severe TBI. The chamber was filled with medical-grade oxygen and pressurized between 1.5 and 2.0 ATA, with a rate of 1-2 psi/min, maintaining an average oxygen concentration of 100%. Treatments were administered up to five times a week, and trained hyperbaric technicians monitored the patient closely for any adverse reactions. After each session, the chamber was depressurized at a rate of 1 to 2 psi/min back to 1.0 ATA [28].

Before treatment, a certified hyperbaric technician (CHT) performed a pretreatment screening, which involved establishing treatment goals, reviewing the patient’s medical history, and discussing potential benefits and risks. The patient was instructed on how to equalize ear pressure, similar to the process of flying on an airplane, to prevent discomfort during the session. She was provided with hospital scrubs to wear and instructed to remove any metal objects, such as jewelry, glasses, dentures, and contact lenses, to avoid damage from the high-pressure oxygen environment [28].

Once prepared, the patient was placed in a chamber filled with pure medical-grade oxygen, which was then sealed. The CHT gradually increased the pressure while maintaining communication with the patient via an intercom system. Treatments lasted between 30 minutes and two hours. After each session, the chamber was slowly depressurized, allowing the patient to exit. Both the patient and caregiver were advised to hydrate and rest before resuming normal activities [28].

Statistical Procedures

IBM SPSS Statistics for Windows, Version 29.0 (Released 2022; IBM Corp., Armonk, NY, USA) was used to perform the PCA. The PCA was carried out on the pretest, posttest, and difference scores following these systematic steps. First, data standardization was applied to ensure that each feature had a mean of zero and a standard deviation of one. A correlation matrix was then computed to clarify the relationships among the variables [32].

To improve the interpretability of the components, a varimax rotation was utilized. This orthogonal rotation technique maximizes the variance of the squared loadings, thereby enhancing the correlation between the original variables and the components. Eigenvalues and eigenvector coefficients were extracted from the correlation matrix to identify the principal components [32]. The eigenvalues were arranged in descending order, and the corresponding eigenvectors were organized accordingly. Only eigenvalues greater than one were retained, resulting in a reduced-dimensional representation of the data that allowed for more manageable and interpretable subsequent analyses. All statistical findings were documented with both textual descriptions and tables to ensure clarity and ease of understanding [32].

Institutional Review

This research study utilized data derived from the integration of HBOT and neurofeedback therapies over a 31-month period, conducted for clinical purposes. The patient consented to participate in this study, with informed consent also obtained from her caregivers for both the neurofeedback and HBOT treatments, as well as for the research utilization of the generated qEEG data. The study neurofeedback and HBOT protocols were submitted to the WIRB-Copernicus Group (WCG®IRB) for review and were granted an exemption (reference #1-1435713). The authors affirm that the treatments and analyses were conducted in strict adherence to the ethical standards delineated in the 1964 Declaration of Helsinki and its subsequent amendments or comparable ethical guidelines.

Results

Brainwave-Specific PCA Pretest Results

The PCA for pretest brainwave activity revealed a 3-factor solution accounting for a cumulative 81.65% of the variation. Factor 1 explained 37.33% of the variation, indicating a predominant dimension capturing the shared variation among the amplitude of brainwave signals. The results of Pretest rotated factor loadings for brainwave-specific results are presented in Table 1.

**Table 1:** Rotated component matrix (PCA) pretest Extraction Method: PCA; Rotation Method: Varimax with Kaiser Normalization; Rotation converged in four iterations (PreTuV2): Pretest-Theta microvolts squared; (PreAuV2): Pretest-Alpha microvolts squared; (PreBuV2): Pretest-Beta microvolts squared; (PreDuV2): Pretest-Delta microvolts squared; (PreDhz): Pretest-Delta hertz; (PreGuV2): Pretest-Gamma microvolts squared; (PreBhz): Pretest-Beta hertz; (PreB2hz): Pretest-Beta2 hertz; (PreB2uV2): Pretest-Beta2 microvolts squared; (PreGhz): Pretest-Gamma hertz; (PreAhz): Pretest-Alpha hertz; (PreThz): Pretest-Theta hertz PCA: principal component analysis

Brainwave-specific variable	Component 1	Component 2	Component 3
(PreTuV2)	0.971	-	-
(PreAuV2)	0.961	-	-
(PreBuV2)	0.947	-	-
(PreDuV2)	0.945	-	-
(PreDhz)	-	0.888	-
(PreGuV2)	0.44	0.849	-
(PreBhz)	-	0.843	-
(PreB2hz)	-	0.787	-
(PreB2uV2)	0.656	0.738	-
(PreGhz)	-	0.43	0.429
(PreAhz)	-	-	-0.844
(PreThz)	-	-	0.813

The patterns observed in the brainwave frequencies in Table 1 may provide insights into the patient's initial neurological state and potential areas for targeted intervention.

Component 1: The significant positive loadings for PreTuV², PreAuV², and PreDuV² suggest that the patient may be exhibiting some restorative potential. High theta and alpha wave activity can indicate that the patient has moments of relaxation and cognitive processing capacity, which are critical for recovery. The strong delta wave presence indicates that the patient may have retained some ability to access deep sleep and restorative states, which are vital for healing after TBI.

Component 2: The significant loading of PreGuV² alongside the presence of PreDhz reflects a potential for higher cognitive functions despite the injury. Gamma waves are often associated with attention and memory, suggesting that cognitive functions might still be accessible. The negative loading for PreAhz could indicate that the patient may be experiencing anxiety or a lack of calmness. This may hinder healing and emotional regulation, signaling a need for therapeutic strategies aimed at promoting relaxation.

Component 3: The presence of PreB2hz and PreB2uV² suggests that there may be periods of hyperarousal or anxiety. While some beta activity is necessary for focus and engagement, excessive beta waves can indicate stress, which is counterproductive for recovery. The loading of PreThz also implies that the patient might oscillate between states of rest and alertness, potentially indicating difficulty in achieving a stable, relaxed state.

The initial PCA results indicate that this TBI patient has retained some cognitive and restorative capacities, but also shows signs of stress and potential anxiety. This duality highlights the complexity of recovery post-TBI and the need for a multifaceted treatment approach to promote healing and improve cognitive function.

Brainwave-Specific PCA Posttest Results

Results of the Posttest PCA for brainwave-specific PCA results revealed a 4-factor solution explaining 82.703% of the total variation in the PCA model. The results of the Posttest rotated factor loadings for brainwave-specific results are presented in Table 2.

**Table 2:** Rotated component matrix (PCA) posttest Extraction Method: PCA; Rotation Method: Varimax with Kaiser Normalization; Rotation converged in seven iterations (PostB2uV2): Posttest-Beta2 microvolts squared; (PostBuV2): Posttest-Beta microvolts squared; (PostTuV2): Posttest-Theta microvolts squared; (PostDuV2): Posttest-Delta microvolts squared; (PostAuV2): Posttest-Alpha microvolts squared; (PostGuV2): Posttest-Gamma microvolts squared; (PostAhz): Posttest-Alpha hertz; (PostB2hz): Posttest-Beta2 hertz; (PostThz): Posttest-Theta hertz; (PostDhz): Posttest-Delta hertz; (PostGhz): Posttest-Gamma hertz; (PostBhz): Posttest-Beta hertz PCA: principal component analysis

Brainwave-specific variable	Component 1	Component 2	Component 3	Component 4
(PostB2uV2)	0.975	-	-	-
(PostBuV2)	0.97	-	-	-
(PostTuV2)	0.969	-	-	-
(PostDuV2)	0.966	-	-	-
(PostAuV2)	0.953	-	-	-
(PostGuV2)	0.889	-	-	-
(PostAhz)	-	-0.809	-	-
(PostB2hz)	-0.449	0.643	-	-
(PostThz)	-	0.562	-	0.468
(PostDhz)	-	-	0.9	-
(PostGhz)	-	0.458	-0.553	-
(PostBhz)	-	-	-	0.901

The PCA results in Table 2 from the posttest data after 31 months of neurofeedback and HBOT reveal noteworthy changes in brainwave frequencies that may provide insights into the patient's recovery and ongoing neurological state.

Component 1: The high loadings for PostB2uV², PostBuV², PostTuV², PostDuV², and PostAuV² indicate a strong presence of multiple brainwave frequencies that are generally associated with cognitive function, alertness, and relaxation. The prominence of beta waves suggests that the patient has developed a more active cognitive state, potentially reflecting improved focus and processing abilities. The sustained presence of theta and delta waves also indicates access to restorative processes, essential for healing, sleep quality, and emotional regulation.

Component 2: The presence of PostAhz with a negative loading, combined with PostB2hz, and PostThz, showing moderate positive loadings, suggests a nuanced interaction between states of calmness and hyperarousal. The negative loading for alpha frequencies could indicate a potential struggle with maintaining relaxation or a tendency toward anxiety. This may suggest that while cognitive function has improved, the patient might still be experiencing challenges in emotional regulation.

Component 3: The strong loading of PostDhz indicates significant activity in this frequency, which is associated with deep sleep and restorative states. This suggests that the patient may have made notable progress in accessing deeper sleep states, contributing to overall recovery. The involvement of PostThz indicates there may be a balance between relaxation and cognitive engagement, which is promising for ongoing rehabilitation.

Component 4: The high loading for PostBhz indicates that while there is an active cognitive state, it may also be associated with heightened alertness or stress. The overall loading patterns suggest that while cognitive and restorative processes have improved, the presence of stress-related beta activity may need further attention.

The lower loadings for PostGhz alongside moderate influences from other components suggest that higher cognitive processing, while improved, might not be fully optimized. This could reflect areas for further cognitive enhancement through targeted therapies.

Overall, the posttest PCA results indicate that the patient has made significant strides in cognitive function and restorative processes after 31 months of neurofeedback and HBOT. However, ongoing challenges related to emotional regulation and potential anxiety indicate areas for further intervention. A balanced approach that continues to promote both cognitive engagement and relaxation will likely be key to the patient's continued recovery.

Brainwave-Specific PCA Difference Score Results

Results of initial difference scores PCA for brainwave-specific PCA indicated a 5-factor solution explaining 89.069% of the total variation in the PCA model. The results of difference score rotated factor loadings for brainwave-specific results are presented in Table 3.

**Table 3:** Rotated component matrix (PCA) difference scores Extraction Method: PCA; Rotation Method: Varimax with Kaiser Normalization; Rotation converged in seven iterations (BhzDIFF): Difference Score Beta Hertz; (GuV2DIFF): Difference Score Gamma microvolts squared; (B2uV2DIFF): Difference Score Beta2 microvolts squared; (DuV2DIFF): Difference Score Delta microvolts squared; (TuV2DIFF): Difference Score Theta microvolts squared; (AuV2DIFF): Difference Score Alpha microvolts squared; (BuV2DIFF): Difference Score Beta microvolts squared; (GhzDIFF): Difference Score Gamma hertz; (AhzDIFF): Difference Score Alpha hertz; (DhzDIFF): Difference Score-Delta hertz; (B2hzDIFF): Difference Score-Beta2 hertz; (ThzDIFF): Difference Score-Theta hertz PCA: principal component analysis

Brainwave-specific variable	Component 1	Component 2	Component 3	Component 4	Component 5
(BhzDIFF)	0.944	-	-	-	-
(GuV2DIFF)	0.928	-	-	-	-
(B2uV2DIFF)	0.925	-	-	-	-
(DuV2DIFF)	-	0.95	-	-	-
(TuV2DIFF)	-	0.937	-	-	-
(AuV2DIFF)	-	-	0.953	-	-
(BuV2DIFF)	0.452	-	0.808	-	-
(GhzDIFF)	-	-	-	0.912	-
(AhzDIFF)	-	-	-	-0.749	-0.507
(DhzDIFF)	-	0.413	-	-0.621	-
(B2hzDIFF)	0.427	-	-	-	0.764
(ThzDIFF)	-	-	0.416	-	0.694

The PCA results from the difference (effect) scores after 31 months of HBOT and neurofeedback treatments indicate a complex interaction of brainwave patterns that may shed light on the recovery process.

Component 1: High loadings for BhzDIFF, GuV²DIFF, B2uV²DIFF, and DuV²DIFF suggest significant improvements in alertness and cognitive processing. The predominance of beta and gamma activity indicates enhanced cognitive function and mental clarity, which are essential for recovery after TBI. The strong correlation among these frequencies suggests a shift toward more organized and functional brain activity, potentially reflecting increased engagement in cognitive tasks and improved mental health.

Component 2: The significant loading of DuV²DIFF and its contribution to this component reflects improved access to restorative processes. Enhanced delta wave activity is crucial for sleep and healing, indicating that the patient may have experienced better sleep quality or more restorative states during recovery. The presence of BuV²DIFF with a slightly lower loading reinforces the idea that while cognitive function is up, there may still be some residual anxiety or hyperarousal, suggesting the need for continued focus on emotional regulation.

Component 3: The significant loading for AuV²DIFF indicates improvements in relaxation and calmness, essential for emotional health and recovery. The presence of this variable alongside others like ThzDIFF shows that the patient is likely achieving a better balance between cognitive engagement and relaxation. The connection between alpha and theta frequencies highlights the potential for creativity and intuitive thought processes, which are vital for rehabilitation.

Component 4: The high loading for GhzDIFF suggests notable gains in higher cognitive functions, including memory, learning, and information processing. This component’s focus on gamma waves reflects significant improvements in cognitive capabilities, which are critical for functional independence. The presence of B2hzDIFF indicates that, while there are gains, there might still be aspects of stress or heightened alertness that need addressing.

Component 5: The negative loadings for AhzDIFF highlight a potential decline in states of calm or increased anxiety. This could indicate that, despite improvements in cognitive function, the patient may still be grappling with emotional challenges, such as anxiety or stress, which could impede overall recovery.

The mixed loadings suggest a complex interplay of cognitive and emotional states, necessitating a tailored approach in ongoing treatment. The PCA of difference scores indicates that the patient has made significant progress after 31 months of treatment, particularly in cognitive function and restorative processes. However, emotional regulation challenges remain, suggesting that ongoing support and tailored interventions are essential for optimizing recovery. A holistic approach that balances cognitive engagement with emotional well-being will likely yield the best outcomes moving forward.

Electrode-Specific PCA Results

The initial Pretest PCA for electrode-specific data revealed a 2-factor solution accounting for 96.321% of the total variation in the PCA model (uV²). Additionally, a 1-factor solution was identified, explaining 99.548% of the total variance in the PCA model (Hz), with only one component extracted, rendering rotation impossible. The pretest data for both uV² and Hz are relatively complex, with two factors for uV² and one factor for Hz, indicating that both dimensions (voltage and frequency) involve more than one component in the pretest phase.

For the initial Posttest PCA, the factor loading for electrode-specific data (uV²) indicated a 1-factor solution explaining 99.559% of the total variation, with a single component extracted and no possibility for rotation. Similarly, the PCA results for electrode-specific data (Hz) showed a 1-factor solution accounting for 99.583% of the total variance, with only one component extracted and no rotation feasible. The posttest data for both uV² and Hz are simpler, with a clear shift to a single factor for both, suggesting that the intervention might have reduced the variability or complexity in the signal, especially in terms of how much variation can be explained by a single component.

The initial Difference Score PCA for electrode-specific data (uV²) demonstrated a 2-factor solution explaining 97.917% of the total variation. In contrast, the Difference Score PCA for electrode-specific data (Hz) revealed a 3-factor solution accounting for 93.439% of the total variation. The difference scores show some increased complexity, particularly in the frequency domain (with a 3-factor solution), which suggests that changes in the frequency domain due to the intervention are multidimensional, whereas changes in the uV² domain are still predominantly explained by two factors.

These results imply that the intervention has affected the data in different ways depending on the domain (uV² vs. Hz). The frequency domain appears to be more sensitive to changes or more complex post-intervention, while the voltage domain (uV²) has simplified post-intervention but still retains some complexity in the difference scores. The absence of rotation in all models reflects that the data is sufficiently explained by the components identified, and no further simplification of the component structure is necessary.

## Discussion

Brainwave-specific qEEG PCA

The results of the PCA conducted on the brainwave activity of a severe TBI patient provide valuable insights into the patient’s neurological state and recovery trajectory following 31 months of neurofeedback and HBOT. The pretest, posttest, and difference score analyses reveal significant changes in brainwave patterns, reflecting both cognitive and emotional aspects of recovery.

HBOT and neurofeedback, when applied to severe TBI, have shown promising potential to address complex neurological symptoms by facilitating neuroplasticity, promoting cellular repair, and modulating brainwave patterns. HBOT provides enhanced oxygen availability to hypoxic or damaged brain areas, supporting tissue repair, reducing neuroinflammation, and promoting angiogenesis, particularly within regions impacted by severe TBI. Neurofeedback, meanwhile, is highly individualized, harnessing real-time qEEG data to help patients learn to modulate their brainwave activity consciously, aligning brain activity with healthier, more functional states [4,28,33,34].

In this secondary analysis, PCA applied to brainwave-specific data reveals increased factor complexity from pretest to posttest, indicating possible neurophysiological adaptations due to the combined neurofeedback and HBOT therapies. The variation explained by each PCA model’s factors may underscore the capacity of HBOT and neurofeedback to catalyze widespread changes, as demonstrated in the 4-factor solution posttest results. The 5-factor solution for difference scores (89.069% variance explained) likely reflects nuanced, individualized therapeutic effects, which align well with neurofeedback’s real-time adaptation to individual brainwave states, allowing for targeted modulation of brainwave-specific domains.

Pretest Analysis 

The pretest PCA identified a 3-factor solution that accounted for 81.65% of the total variation, indicating a robust structure in the patient’s brainwave data. Component 1’s high loadings of theta, alpha, and delta waves suggest the patient exhibited restorative potential, which is crucial for recovery. Specifically, elevated theta and alpha activity is associated with relaxation and cognitive processing, while strong delta wave presence points to the retention of deep sleep capabilities. These states are essential for healing, as they facilitate neuroplasticity and emotional regulation [35-42].

The negative loading for alpha frequency indicates potential anxiety, which could hinder recovery efforts. The presence of beta2 activity suggests periods of hyperarousal or stress, potentially complicating the healing process. The findings underscore the complexity of recovery post-TBI, highlighting the need for multifaceted therapeutic interventions that address both cognitive enhancement and emotional well-being [35-42].

Posttest Analysis

The posttest PCA revealed a 4-factor solution explaining 82.703% of the total variation, indicating a marked improvement in the patient’s brainwave dynamics. The predominance of beta and gamma activity in Component 1 suggests enhanced cognitive function and alertness, vital for functional independence following TBI. The sustained presence of theta and delta waves reinforces the idea of improved restorative processes, which are essential for emotional and physical recovery.

These PCA findings coincide with the post-treatment qEEG conducted in June 2024 (Peterson et al.’s initial study) which found moderate to large improvements in the patient’s brain’s average frequency band parameters (g = 0.612) and small to moderate effects on 19 electrode placement outcomes (uV² g = 0.339 and Hz g = 0.333) [28]. These June 2024 (initial study) results reflect significant progress over a 31-month treatment period. Objective measures, including the DRS and the GOSE, showed substantial improvements in cognitive abilities such as feeding (p = 0.046), toileting (p = 0.046), grooming (p = 0.046), and communication skills (p = 0.046). According to the qEEG effect sizes, as well as DRS and GOSE scores from pretest (2021) and posttest (2024), the patient demonstrated meaningful gains in brain recovery and overall quality of life [28].

Also, the negative loading for alpha frequency in Component 2 suggests ongoing challenges with anxiety and relaxation, despite cognitive improvements. This highlights the dual nature of recovery, where cognitive function may advance while emotional regulation requires further intervention. The significant loading of delta waves indicates a positive trend towards accessing deeper sleep states, essential for comprehensive healing [35-42].

Difference Score Analysis

The PCA of the difference scores provided a 5-factor solution, explaining 89.069% of the total variation. These results underscore the dynamic interplay between cognitive and emotional states during the recovery process. The strong loadings in Component 1 for beta and gamma waves reflect significant enhancements in cognitive clarity and engagement, critical for rehabilitation efforts. The observed improvements in delta wave activity (Component 2) further emphasize the patient’s progress in achieving restorative sleep, a key factor in recovery [35-42].

The presence of negative loadings for alpha frequencies in Component 5 indicates that emotional challenges persist, suggesting that despite cognitive gains, the patient may still be navigating heightened anxiety or stress. This duality of progress emphasizes the need for targeted therapeutic strategies to promote emotional well-being alongside cognitive enhancements [35-42].

The PCA results across pretest, posttest, and difference scores illuminate the complex nature of recovery in a severely TBI patient. While significant strides have been made in cognitive function and restorative processes, ongoing emotional regulation challenges indicate the need for comprehensive, individualized treatment plans. A balanced approach that integrates cognitive training with strategies for managing anxiety and promoting relaxation will likely optimize recovery outcomes. Future research should explore the longitudinal effects of neurofeedback and HBOT, aiming to refine therapeutic protocols that address the multifaceted aspects of TBI recovery [35-42].

Electrode-specific qEEG PCA

*Pretest (Electrode-Specific, μV²)* 

PCA for electrode-specific results in pretest μV² values reveals a two-factor solution, with Factor 1 explaining 84.93% of the variance. High loadings across almost all electrodes on the first component suggest a broadly distributed activation pattern, likely capturing general power in μV² across the scalp. Factor 2 explains 11.39% of the variance, with strong loadings on Fp1 and Fp2 electrodes, indicating potential differential activity in the frontal regions, which are often associated with executive functions and attention [35-42].

*Pretest (Electrode-Specific, Hz)* 

The frequency-specific data yields a single-factor solution that explains nearly 100% of the variance, with high loadings across all electrodes. This uniform pattern suggests minimal spatial differentiation across frequencies in the pretest condition, perhaps indicating baseline homogeneity across the scalp [35,36].

Baseline homogeneity across the scalp in electrode-specific qEEG can indicate a brain-injured state. This homogeneity often reflects a lack of normal variability in brainwave patterns, which is typical in healthy brains. In cases of brain injury, such as TBI, the brain’s electrical activity can become more uniform due to damage to neural networks and reduced functional connectivity [33-42].

Studies have shown that TBI can lead to decreased variability in EEG power across different brain regions, which is often associated with poorer outcomes. This uniformity might be due to the brain’s impaired ability to generate diverse and complex electrical signals, which are necessary for normal cognitive and physiological functions [33-42].

Posttest (Electrode-Specific, μV²) 

Similar to the pretest condition, the posttest data for μV² also produces a single-factor solution accounting for 99.56% of the variance. This high variance explained by a single component suggests that post-intervention brain activity, as measured by μV², exhibits coherent changes across the scalp. The lack of distinct regional components implies widespread effects of the intervention across all brain regions, which may be indicative of a global neurophysiological effect [35-42]. 

*Posttest (Electrode-Specific, Hz)* 

The posttest frequency data similarly yielded a single component, explaining 99.58% of the variance. This finding reinforces the interpretation that the intervention produced global changes in frequency, affecting all scalp regions uniformly [35-42].

*Difference Scores (Electrode-Specific, *uV²)

PCA results for electrode-specific difference scores provides a more granular view of how the intervention effects brain activity across specific brain regions. By analyzing PCA loadings associated with individual electrodes, we can identify which brain areas show the most substantial changes in power and frequency following the intervention [35-42].

Difference scores (effect scores) in microvolts squared (uV²)

Component Loadings by Electrode

The five components (5-Factor solution) in the uV² PCA capture distinct clusters of power changes associated with different brainwave bands. Examining the loadings for each electrode within these components provides insight into regional effects.

Component 1 (High Beta and Gamma Power)

High loadings on Beta (Bhz) and Gamma (GuV²) power changes, as well as Beta2 power (B2uV²), suggest that electrodes in areas associated with cognitive processing and executive function (such as frontal and central regions, e.g., Fz, Cz) may show significant increases in higher-frequency power. This could reflect increased activity in the prefrontal cortex or sensorimotor cortex, areas typically linked to attention, problem-solving, and motor control [35-42].

Component 2 (Delta and Theta Power)

Loadings on Delta (DuV²) and Theta (TuV²) power changes suggest that parietal and occipital electrodes (e.g., Pz, Oz) may show prominent changes in these low-frequency bands, which are often associated with restfulness and meditative states. Changes here could indicate that the intervention impacts regions involved in relaxation and the default mode network, potentially enhancing relaxation or recovery-related processes [35-42].

Component 3 (Alpha Power)

This component, which loads on Alpha power (AuV²), may be driven by electrodes located over midline and occipital regions (e.g., Cz, Oz), where Alpha activity is traditionally most prominent. Since Alpha is associated with calm wakefulness, this suggests that these regions may be experiencing enhanced relaxation or reduced cortical excitability, possibly leading to a greater ability to disengage from external stimuli post-intervention [35-42].

Component 4 (Gamma Frequency Power)

High loadings on Gamma (Ghz) indicate that frontal and central electrodes (e.g., Fz, FCz, Cz) might be particularly affected in terms of high-frequency Gamma power. This could signify enhanced cognitive processing or increased connectivity in these areas, reflecting possible improvements in cognitive flexibility and working memory functions [35-42].

Component 5 (Beta2 and Theta Frequency)

Moderate loadings on Beta2 (B2hz) and Theta (Thz) suggest that electrodes over temporal and parietal regions (e.g., T7, T8, P3, P4) may show a nuanced interaction between high- and low-frequency power changes, possibly indicating improvements in attentional control and internal processing [35-42].

Electrode-specific results for difference scores (effect scores) in Hertz (Hz)

For the Hertz (Hz) PCA, the single component with 99.58% of the variance indicates a uniform shift in frequency across nearly all electrodes. However, analyzing the minor nuances in the loadings may still highlight areas of slight variability, offering clues to regional effects.

Global Frequency Shift

The uniformity of this single component suggests that almost all electrodes experience similar frequency changes across bands, indicating a global realignment of brain oscillations. This effect could imply enhanced synchronization across brain regions, supporting improved coherence and connectivity as a whole [35-42].

While all electrodes participate in this uniform shift, slight variations may still emerge, with frontal (e.g., Fz) and central electrodes (e.g., Cz) potentially showing slightly stronger frequency shifts. This would align with areas often involved in higher-order processing and executive functions, where coherent oscillatory shifts could reflect enhanced cognitive integration post-intervention [35-42].

Summary of electrode-specific findings

Microvolts Squared (uV²)

Power changes are distributed across electrodes associated with specific components, highlighting a more localized effect of the intervention on brain power. Frontal and central electrodes are more involved in high-frequency Beta and Gamma changes, associated with cognitive processing, while occipital and parietal electrodes show shifts in low-frequency Delta and Theta bands, which are associated with relaxation [35-42].

Hertz (Hz)

The single component indicates a nearly global frequency shift across electrodes, suggesting a pervasive synchronization effect. Minor electrode-specific variations may highlight slight enhancements in frontal-central regions, aligning with improved executive functions and integration [35-42].

Together, the electrode-specific results suggest that the intervention drives both local and global changes in brain activity. It appears to promote high-frequency power changes in regions tied to cognitive functions (e.g., frontal and central regions) while supporting low-frequency power changes in areas associated with rest and relaxation (e.g., parietal and occipital). At the same time, the broad, uniform frequency shift across electrodes highlights a global synchronizing effect, possibly facilitating enhanced neural coherence across the brain. This combined impact may underlie the therapeutic benefits observed, supporting both localized and widespread improvements in brain function [35-42].

Limitations

Examining the therapeutic impact of combined HBOT and neurofeedback on TBI, several inherent limitations exist, which must be carefully considered to contextualize findings. Here are the primary limitations, elaborated.

The complexity of TBI pathology with severe TBI presents with highly heterogeneous symptoms and injury profiles across patients, given the individualized nature of brain injury location, severity, and related neurophysiological impairments. The varying structural and functional damages make it challenging to attribute neurophysiological improvements directly to the interventions, as some symptom changes may stem from factors like spontaneous recovery or compensatory brain reorganization.

PCA, while beneficial for reducing dimensionality and identifying patterns within qEEG data, is complex and can lead to ambiguous interpretations. PCA-derived factors may not have inherent biological or functional meanings, and rotation choices (e.g., varimax rotation) can affect factor loadings and interpretation. This reliance on data-driven factor identification makes it difficult to draw definitive conclusions about specific brainwave frequency or electrode site changes, especially when translating them into clinical outcomes or specific neurophysiological adaptations.

qEEG captures only certain aspects of brain function (mainly cortical electrical activity) and may not reflect deeper brain changes or more subtle biochemical processes. Additionally, artifact contamination, such as muscle activity or eye movements, can obscure the data. While neurofeedback protocols often focus on targeted brainwave modulations, they may not address deeper structures that also contribute significantly to TBI symptoms.

Also, without a comparison group receiving a placebo or alternate therapy, attributing the observed changes solely to the combined intervention remains speculative. A comparison group would allow for direct comparisons to rule out alternative explanations, such as placebo effects, spontaneous recovery, or the effects of increased patient engagement.

Studies that involve specific combinations of HBOT and neurofeedback are challenging to generalize due to factors like varying HBOT protocols (e.g., duration, pressure, oxygen concentration) and neurofeedback protocols (e.g., targeted brainwaves and electrode sites). The individualized nature of neurofeedback, with adjustments based on real-time feedback, means results may not uniformly apply to other settings or populations with different neurofeedback or HBOT configurations.

The intervals between pretest and posttest measurements (November 2021 to June 2024), and the overall timing of intervention effects, may not align with the timeline of neural adaptation. While some neurophysiological adaptations may appear immediately, others may emerge over longer periods, creating potential discrepancies between the timing of measurements and the actual trajectory of recovery or symptom change. Follow-up studies with more extended monitoring periods are essential to capture long-term effects.

A study with a limited sample size, especially with advanced statistical methods like PCA, can lead to overfitting, where the model captures noise or idiosyncratic data patterns rather than true population-wide effects. In repeated measures designs, this can introduce further limitations if individual variations drive factor solutions or apparent improvements rather than genuine intervention effects.

Studies combining HBOT and neurofeedback often attract participants with high expectations for recovery, which can introduce bias through patient self-report measures. Additionally, researchers may have difficulty maintaining a fully blinded approach, which may unconsciously influence data interpretation, particularly in exploratory analyses.

The synergistic effects of combining HBOT and neurofeedback are not yet fully understood, raising ethical concerns around unanticipated side effects or interactions between therapies. As each therapy impacts neurophysiological systems in unique ways, combining them may amplify risks in unknown areas, necessitating rigorous risk-benefit assessments to ensure patient safety.

Addressing these limitations is crucial for the robustness of conclusions in HBOT and neurofeedback research for TBI. Future studies that incorporate control groups, larger sample sizes, extended monitoring, and randomized protocols would help mitigate these challenges and improve the validity and generalizability of findings.

## Conclusions

PCA factor structure results indicate noteworthy baseline and post-intervention differences across brainwave frequencies and electrode locations. The pretest data’s simpler factor structure evolves into a more complex, multifactor solution in posttest and difference score analyses, likely reflecting neurophysiological adaptations due to the therapeutic intervention. Notably, the five-factor solution for difference scores may represent nuanced, individualized therapeutic effects, highlighting distinct functional domains impacted by the intervention across different brainwave frequencies and scalp locations. These findings may underscore the potential of neurofeedback and HBOT to induce widespread and specific neurophysiological changes.

The combined use of HBOT and neurofeedback may show promise in treating severe TBI by promoting neurogenesis, reducing inflammation, enhancing neuroplasticity, and improving cognitive and emotional functions. The PCA results may begin to provide a detailed understanding of the factor structure relative to neurophysiological changes, demonstrating the HBOT and neurofeedback interventions’ capacity to induce significant and individualized brain recovery. These findings might highlight the potential of these therapies to improve the quality of life for individuals with severe TBI, offering hope for more effective treatment strategies in the future.
