# Correction: Long-Term Effects of Neurofeedback and Hyperbaric Oxygen Therapy on Traumatic Brain Injury: A Principal Component Analysis (PCA)-Based Secondary Analysis

**DOI:** 10.7759/cureus.c244

**Published:** 2025-08-13

**Authors:** Tami Peterson, JeAnnah AbouAssaly, Sheila Burgin, Robert Sherwin, Frederick Strale

**Affiliations:** 1 Hyperbaric Oxygen Therapy, The Oxford Center, Brighton, USA; 2 Neurofeedback, The Oxford Center, Brighton, USA; 3 Hyperbaric Oxygen Therapy, Wayne State University School of Medicine, Detroit, USA; 4 Biostatistics, The Oxford Center, Brighton, USA

The Conflicts of Interest statement has been updated to declare the following:

Financial relationships: Tami Peterson, JeAnnah AbouAssaly, Sheila Burgin, Robert Sherwin and Frederick Strale Jr. declare employment from The Oxford Center. 

